# In Vitro Pre-Cecal Digestion and Cecal Degradation of Pea Hulls, Artichoke Bracts, Date Flesh, and Date Pits for Rabbit Nutrition

**DOI:** 10.3390/ani16203275

**Published:** 2026-10-17

**Authors:** Sonia Tassone, Khalil Abid, Hatsumi Kaihara, Seifeddine Ben Rejeb, Takwa Abidi, Samia Ben Said, Wijden Niama, Mohamed Aroua, Mokhtar Mahouachi

**Affiliations:** 1 Department of Agricultural, Forest and Food Sciences, University of Turin, Largo P. Braccini 2, 10095 Grugliasco, Italy; sonia.tassone@unito.it; 2 ZeroMPs S.r.l. Via Adige 9, 10098 Rivoli, Italy; 3 Ecole Supérieure d’Agriculture du Kef, University of Jendouba, Le Kef 7119, Tunisia; mrsaifddinebenrajeb@gmail.com (S.B.R.); abiditakwa506@gmail.com (T.A.); sabensaid@univjendouba.tn (S.B.S.); naaymawejden@gmail.com (W.N.); arouamohamed2310@gmail.com (M.A.); taymallahmah@gmail.com (M.M.)

**Keywords:** co-products, rabbit nutrition, pre-cecal digestibility, cecal degradation, pea hulls, artichoke bracts, date pits, date flesh, sustainable feed resources

## Abstract

Rising feed costs and the need for more sustainable livestock production have increased interest in co-products as alternative feed resources for rabbits. However, their nutritional value may be underestimated when evaluated only through pre-cecal enzymatic digestion, without considering cecal degradation. This study comparatively characterized pea hulls, artichoke bracts, date flesh, and date pits (three independent sources each, *n* = 3) and evaluated their nutritional potential using a sequential in vitro procedure combining pre-cecal enzymatic digestion with cecal degradation. Date flesh, characterized by high non-fibrous carbohydrate and low fiber, showed the highest pre-cecal enzymatic dry matter and organic matter digestibility (89.44% and 89.58%, respectively), with minimal additional contribution from cecal degradation. Pea hulls contained the highest crude protein (24.97% of dry matter) and showed high additional degradation of structural carbohydrates during the cecal phase, reaching cumulative neutral detergent fiber and acid detergent fiber digestibility (47.18% and 41.90%, respectively). Artichoke bracts showed moderate pre-cecal enzymatic digestibility and substantial additional utilization during cecal degradation. Date pits had the highest fiber, lignin, and tannin concentrations and the lowest pre-cecal enzymatic digestibility, although cecal degradation partially improved their nutrient utilization. Overall, these in vitro results suggest that cecal degradation can contribute substantially to the digestibility of residual structural carbohydrates in fiber co-products.

## Simple Summary

The increasing cost of feed and the need to reduce agricultural waste have created interest in using co-products as alternative feed ingredients for rabbits. However, the nutritional value of these materials may be underestimated when only enzymatic digestion is considered, because rabbits can also use nutrients through microbial fermentation. This study evaluated four co-products (pea hulls, artichoke bracts, date flesh, and date pits) to determine their composition and their potential digestive utilization in rabbits, estimated with laboratory tests. The results showed clear differences among the four co-products. Date flesh was rich in available carbohydrates and showed a very high in vitro digestibility, with almost 90% of its dry and organic matter digestibility during simulated digestion, before the cecal stage. Pea hulls contained the highest amount of protein and showed substantial digestibility of their fiber during cecal incubation. Artichoke bracts also provided useful fiber and protein, with additional nutrient digestibility during cecal incubation. Date pits contained very high levels of fiber and tannins and had the lowest initial digestibility, although cecal incubation increased it. These findings suggest that considering both pre-cecal enzymatic digestion and cecal degradation gives more complete information on the potential nutritional value of these co-products. Their use could help reduce waste and support more sustainable rabbit production.

## 1. Introduction

The valorization of co-products as alternative feed resources represents a promising strategy to improve the sustainability of livestock production [1,2,3]. Their use may contribute to recovering valuable nutrients and bioactive compounds, reducing reliance on conventional feed ingredients, and limiting waste disposal while improving resource-use efficiency within a circular bioeconomy framework [1,2].

This approach is particularly relevant in regions where climatic conditions, limited feed availability, high costs of conventional ingredients, and dependence on imported feed materials limit productivity and challenge the sustainability of livestock production [1,4]. Under these conditions, locally available agricultural and food-processing co-products may provide complementary feed resources while creating value from underutilized biomass and promoting more resource-efficient and circular production systems [1,4].

Tunisia’s agricultural and food-processing sectors generate crop residues and co-products with potential for feed valorization. Pea hulls, artichoke bracts and stems, and date-processing co-products are of particular interest, given the country’s production of 52.4 × 10^3^ t of green peas, 25.4 × 10^3^ t of artichokes, and 400.2 × 10^3^ t of dates in 2024 [5]. Pea hulls account for approximately 10–14% of total pea mass [6], while non-edible artichoke bracts and stems may represent approximately 15–30% of artichoke biomass [7]. Low-grade dates and date pits have account for 10–15% of total date production [8]. These co-products may retain nutritionally valuable fractions, including structural carbohydrates, protein, readily available carbohydrates, and bioactive compounds, supporting their potential valorization as alternative feed resources [2,3].

In this context, rabbits (*Oryctolagus cuniculus*) are particularly suitable for the valorization of heterogeneous agro-industrial feed resources because their digestive physiology enables the utilization of fibrous and unconventional feed ingredients [2,9]. As hindgut fermenters, rabbits possess a highly developed cecum hosting a complex microbial community. Nutrients escaping gastric and small-intestinal digestion, particularly fermentable fiber, undergo microbial fermentation in the cecum, resulting in volatile fatty acids as major fermentation end-products [9,10]. Cecotrophy further contributes to nutrient utilization through the reingestion of soft feces, allowing the recovery of microbial protein and other nutrients derived from cecal degradation [9,10]. Consequently, the feeding value of fibrous co-products cannot be assessed solely on the basis of enzymatic digestibility, as microbial fermentation of residual fractions reaching the cecum may provide an additional contribution to their overall digestive utilization [2,10].

Despite the growing interest in co-products as alternative feed resources, comparative information on their sequential digestive utilization in rabbits remains limited, particularly regarding the contribution of cecal degradation to nutrients escaping gastric and small-intestinal digestion [2]. This issue is especially relevant when comparing substrates differing substantially in nutrient composition, cell-wall concentration, lignification, and tannin content. Considering both digestive phases may therefore provide a more comprehensive assessment of heterogeneous co-products. The novelty of the present study lies in sequential assessment of pre-cecal enzymatic digestibility followed by cecal degradation.

In vitro techniques represent practical alternatives to in vivo methods for the nutritional evaluation of feedstuffs, as they allow multiple substrates to be assessed simultaneously under standardized and replicated experimental conditions without the need for experimental animals, thus addressing ethical considerations [11,12]. These approaches also facilitate the comparative characterization of feed ingredients with heterogeneous chemical compositions by providing controlled conditions for investigating specific aspects of digestive utilization. In rabbit nutrition, enzymatic methods can be used to simulate the digestive processes occurring before the cecum, whereas cecal inoculum-based techniques allow the subsequent microbial degradation of undigested residues to be investigated. The sequential application of these methods therefore provides an opportunity to distinguish between the fraction of nutrients digested during the pre-cecal phase and the additional fraction degraded through cecal microbial activity, thereby offering a more comprehensive assessment of substrate utilization. Previous studies have compared enzymatic and cecal inoculum-based methods with in vivo digestibility measurements in rabbits, supporting their use as practical tools for the comparative evaluation of feedstuffs [11,12].

Accordingly, the present study aimed to comparatively characterize the nutritional composition of four co-products collected in Tunisia (pea hulls, artichoke bracts, date flesh, and date pits) and to evaluate their in vitro pre-cecal enzymatic, cecal degradation and cumulative (pre-cecal enzymatic + cecal) digestibility coefficients. By comparing their nutritional and digestive profiles, the study provides in vitro information on their potential as locally available feed resources, thereby contributing to their valorization within more sustainable and circular rabbit production systems.

## 2. Materials and Methods

### 2.1. Co-Products and Sample Preparation

Four co-products generated by Tunisian processing industries were selected for the present study: pea hulls (*Pisum sativum* L.), artichoke bracts (*Cynara scolymus* L.), date flesh, and date pits (*Phoenix dactylifera* L.). Samples were collected directly from three independent industrial processing facilities for each co-product during the 2024–2025 agricultural season. Each co-product was collected from three producers and considered biological replicates (*n* = 3), thereby accounting for variability associated with their origins. For the chemical composition, the three independent sources of each co-product represent the biological replicates (*n* = 3).

Immediately following collection, the samples were oven-dried at 60 °C for 48 h, and ground to pass through a 1-mm screen. The resulting powders were thoroughly homogenized and stored in sealed containers under dry conditions at room temperature until analysis.

### 2.2. Chemical Analyses

The chemical analyses were performed in triplicate on the three independently collected samples of each of the four co-products.

Dry matter (DM), ash, crude protein (CP), and ether extract (EE) were determined according to standard AOAC procedures [13]. DM was determined by oven drying at 105 °C for 5 h, while ash was determined by incineration at 550 °C for 5 h, CP by the Kjeldahl method, and EE by Soxhlet extraction.

Organic matter (OM) and non-fibrous carbohydrates (NFC) were calculated according to AOAC [13]. OM was calculated as the difference between DM and ash contents, whereas NFC was estimated by difference according to the following equation:

NFC = 100 − (NDF + EE + CP + Ash)

where NFC, NDF, EE, CP, and ash were expressed as percentages of DM.

Fiber fractions were analyzed according to Van Soest et al. [14]. Neutral detergent fiber (NDF) and acid detergent fiber (ADF) were determined using an ANKOM200 Fiber Analyzer (ANKOM Technology, Macedon, NY, USA), whereas acid detergent lignin (ADL) was determined using a Daisy^II^ system. Hemicellulose and cellulose were calculated as the difference between NDF and ADF, and ADF and ADL, respectively.

### 2.3. Determination of Tannins

Sample extracts for tannin assays were prepared as follows: 1 g of ground sample (1-mm) was extracted with 20 mL of water, for 24 h at 26 °C by maceration. The aqueous extracts were then filtered through a Whatman No. 1 filter paper and stored in the dark at 4 °C until analysis. Condensed tannins were determined using the vanillin–HCl colorimetric assay described by Julkunen-Tiitto [15]. Briefly, 50 µL of sample extract was mixed with 1500 µL of 4% vanillin solution prepared in methanol, followed by the addition of 750 µL of concentrated HCl. The reaction mixture was incubated in the dark for 20 min, and absorbance was subsequently measured at 550 nm. Condensed tannin concentration was quantified using catechin as the calibration standard over a concentration range of 0–1000 µg/mL. The results were corrected for the dilution factor and expressed as g catechin equivalents per 100 g of dry matter (g CE/100 g DM) [15]. Total tannins were determined using a Folin–Ciocalteu-based colorimetric procedure described by Kujala et al. [16]. Briefly, 500 µL of sample extract was mixed with 500 µL of 50% Folin–Ciocalteu reagent and incubated for 5 min. Subsequently, 1000 µL of 20% sodium carbonate solution was added, followed by a further 10-min incubation. The samples were then centrifuged at 1000× *g* for 5 min, and the absorbance of the resulting supernatant was measured at 730 nm. Total tannin concentration was determined using tannic acid as the calibration standard, and the results were expressed as g tannic acid equivalents per 100 g of dry matter (g TAE/100 g DM) [16].

### 2.4. In Vitro Digestibility Assay

In vitro digestibility was assessed using a sequential procedure combining simulated pre-cecal enzymatic digestion and cecal degradation. All analytical samples (*n* = 120 per run, 240 in total) were first subjected to a three-step enzymatic pre-cecal digestion involving pepsin, pancreatin, and Viscozyme, following the procedure described by Tassone et al. [11]. After pre-cecal enzymatic digestion, one set of Ankom F57 filter bags (ANKOM Technology, Macedon, NY, USA; nominal pore size: 25 µm) (*n* = 120 in total) was used to determine pre-cecal enzymatic digestibility, while a second set (*n* = 120 in total), containing the pre-cecal enzymatically undigested residues, was further subjected to cecal incubation according to Pascual et al. [12], allowing determination of cumulative pre-cecal enzymatic-plus-cecal digestibility.

The digestion experiment was performed in two independent incubation runs, both conducted by the same operator under identical procedures to minimize operator-related variability. For each incubation run, fresh cecal contents were collected from 10 healthy adult male New Zealand White rabbits (*Oryctolagus cuniculus*), approximately 2.5 months of age, with a mean body weight of approximately 4 kg. The animals were sourced from a commercial slaughterhouse (Azienda Agricola Scaglia, Rivoli, Turin, Italy) during routine rabbit meat production. All animals passed ante- and post-mortem veterinary inspections and were considered clinically healthy at the time of slaughter. Before slaughter, the rabbits were maintained under comparable husbandry and feeding conditions and received a commercial pelleted diet *ad libitum* for 42 days before slaughter. The diet contained 15.5% crude protein, 2.1% ether extract, 25.3% neutral detergent fiber, and 6.3% ash. Feed was withdrawn for 8 h before slaughter. No antimicrobial treatments had been administered to the animals, either directly or through feed or drinking water. Cecal contents were collected immediately after slaughter in a thermos flask pre-warmed at 39 °C and saturated with CO_2_. The contents were pooled in equal proportions to prepare the microbial inoculum and transported to the laboratory within 20 min of slaughter. The pH of the pooled cecal contents was 6.1 ± 0.1 in both incubation runs. The pH of the pooled cecal contents was measured using a calibrated portable pH meter (Orion Star A221, Thermo Scientific, Montreal, QC, Canada).

**Figure 1:**
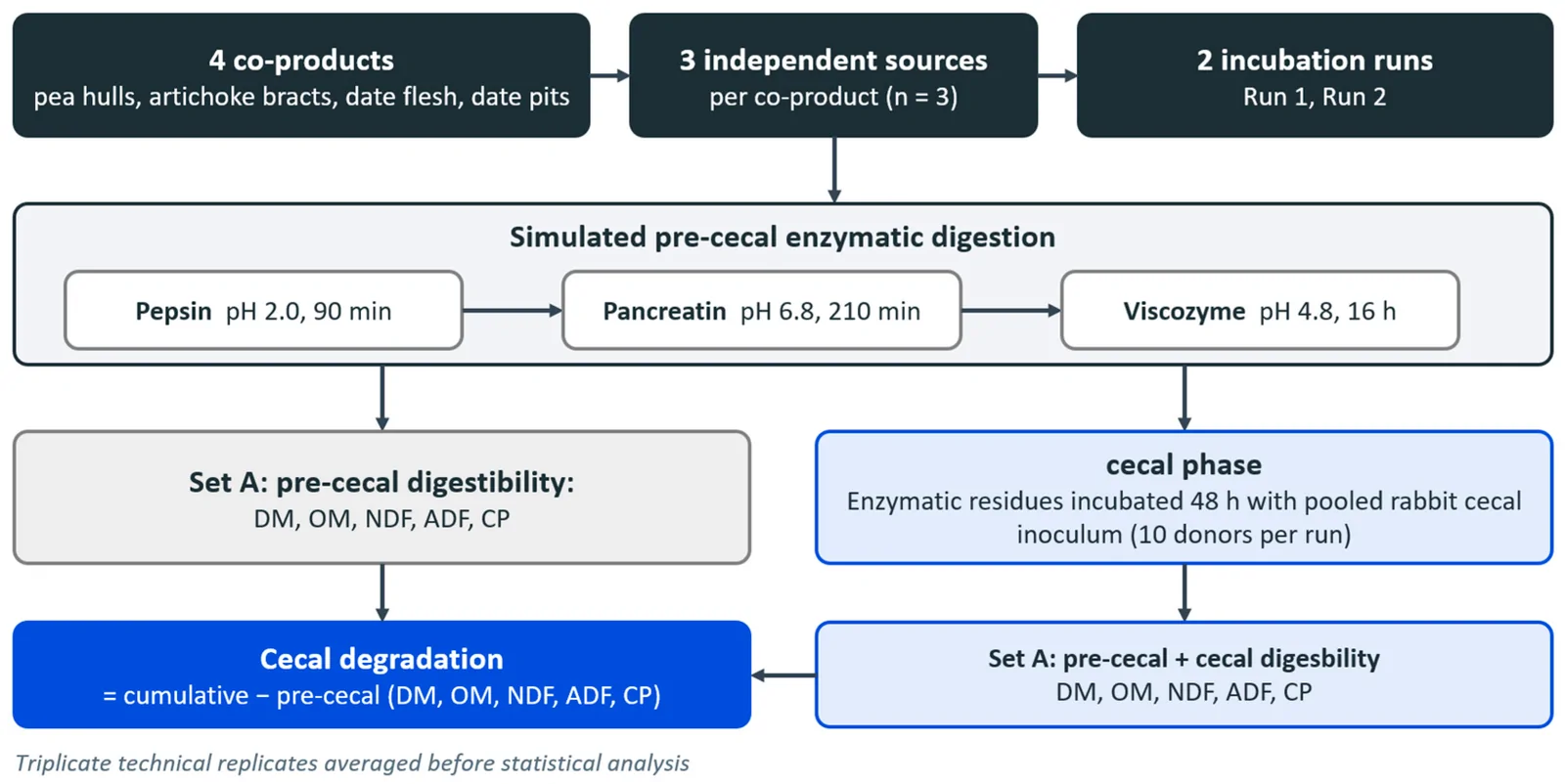
Experimental design of in vitro digestibility. DM, dry matter; OM, organic matter; CP, crude protein; NDF, neutral detergent fiber; ADF, acid detergent fiber.

As the animals were slaughtered exclusively for commercial purposes and were not specifically reared or euthanized for this study, no experimental procedures or interventions were performed on them. The two incubation runs served as experimental replicates, while the three independent sources of each co-product were considered biological replicates within the experimental treatments. The experimental design of the in vitro incubation study is schematically illustrated in Figure 1. The cecal contents of the 10 rabbits collected for each run were pooled; therefore, individual rabbits were not treated as replicates, and the pooled inoculum of each run represents an inoculum batch common to all co-products. The same three independent sources of each co-product were incubated in both runs.

#### 2.4.1. In Vitro Pre-Cecal Enzymatic Digestion

Pre-cecal enzymatic digestion was performed using two Daisy^II^ Incubators (ANKOM Technology, Macedon, NY, USA), each equipped with four digestion jars, with one jar assigned to each co-product. Within each jar, three sets of Ankom F57 filter bags were prepared for each biological replicate: one for the sequential determination of DM and OM, one for the sequential determination of NDF and ADF, and one for CP determination. Each biological replicate was analyzed in triplicate within each set, and one blank was included for each set. Thus, each jar contained 30 Ankom F57 filter bags (27 sample-containing bags and three blanks), resulting in 120 Ankom F57 filter bags per incubator and 240 Ankom F57 filter bags for the entire experiment. Each Ankom F57 filter bag, pre-treated with acetone for 10 min and oven-dried at 105 °C for 24 h, contained 0.50 ± 0.05 g of ground sample. All steps and reagents of the digestion procedure were similar to Tassone et al. [11].

The gastric phase was carried out in 600 mL of 0.1 M phosphate buffer (pH 6.0) and 240 mL of 0.2 M HCl, with the pH adjusted to 2.0 using 1 M HCl or NaOH. After equilibration for 30 min at 40 °C, 0.6 g of pepsin from porcine gastric mucosa (Emprove^®^ exp, 2000 FIP-U/g, EC 3.4.23.1; Merck KGaA, Darmstadt, Germany; cat. no. 1.07190.1000, lot K48188190 634) was added, corresponding to 1200 FIP-U per jar (≈89 FIP-U/g substrate). The Ankom F57 filter bags were incubated for 90 min at 39 °C.

For the pancreatic phase, 240 mL of 0.2 M phosphate buffer (pH 6.8) and 120 mL of 0.6 M NaOH were added, and the pH was adjusted to 6.8 using 1 M HCl or NaOH. Pancreatin from porcine pancreas (4 × USP specifications, i.e., ≥100 USP U amylase, ≥8 USP U lipase and ≥100 USP U protease per mg; Sigma-Aldrich, St. Louis, MO, USA; cat. no. P1750-500G, lot SLBN9076V) (2.4 g) was then added, corresponding to 240,000 U amylase, 19,200 U lipase and 240,000 U protease per jar (≈17,800 U amylase, 1420 U lipase and 17,800 U protease per g of substrate). The Ankom F57 filter bags were incubated for 210 min at 39 °C.

For the third enzymatic phase, the pH was adjusted to 4.8 using 30% acetic acid, and 12 mL of Viscozyme^®^ L, a multi-enzyme complex from *Aspergillus aculeatus* (≥100 FBGU/g; Sigma-Aldrich, St. Louis, MO, USA; cat. no. V2010-250ML; lot SLCL2312; Viscozyme is a registered trademark of Novozymes Corp.) was added, corresponding to ≈1440 FBGU per jar (≈107 FBGU/g substrate). The Ankom F57 filter bags were incubated for 16 h at 39 °C. At the end of the pre-cecal enzymatic procedure, the digestion medium was discarded, and the Ankom F57 filter bags were sequentially rinsed with distilled water, 95% ethanol for 5 min, and acetone for 5 min.

Following pre-cecal enzymatic digestion, 120 Ankom F57 filter bags were used to determine pre-cecal enzymatic digestibility, whereas the remaining 120 Ankom F57 filter bags, containing the pre-cecal enzymatically undigested residues, were subsequently subjected to cecal degradation to calculate cumulative (pre-cecal enzymatic + cecal) digestibility. The 120 Ankom F57 filter bags used to determine in vitro pre-cecal enzymatic digestibility were dried at 60 °C for 24 h to remove residual moisture. This drying procedure was intended to minimize moisture-related changes in the residual material during subsequent handling and analysis while limiting excessive thermal exposure that could potentially alter the chemical composition of the residues. Following drying, residual DM, OM, and CP were determined according to AOAC procedures [13], whereas NDF and ADF were determined according to Van Soest et al. [14]. For the DM and OM set, residual DM was determined first, and the same residues were subsequently used for OM determination. Similarly, the NDF and ADF set were used sequentially for NDF and ADF determination, whereas a separate set was used for CP analysis. All residual values were corrected using the corresponding blanks. Pre-cecal enzymatic digestibility was calculated from the disappearance of each component during enzymatic digestion, based on the difference between its initial and blank-corrected residual amounts, following Tassone et al. [11].

#### 2.4.2. In Vitro Cecal Degradation

In vitro cecal degradation was evaluated using the remaining enzymatically undigested residues (*n* = 120 Ankom F57 filter bags) obtained after the three-step enzymatic digestion. The procedure was based on Pascual et al. [12] and adapted according to Tassone et al. [11]. Briefly, 200 g of pooled cecal contents was homogenized with 320 mL of artificial saliva under a continuous CO_2_ to maintain anaerobic conditions and preserve microbial activity. The artificial saliva contained 0.5 g/L MgSO_4_·7H_2_O, 1.5 g/L NaCl, 3.8 g/L NaHCO_3_, 4.0 g/L K_2_HPO_4_, and 0.5 g/L (NH_4_)_2_HPO_4_. The prepared inoculum was maintained under anaerobic conditions for 1 h, after which the liquid fraction was recovered by decantation without centrifugation. The recovered inoculum was transferred to digestive jars containing Ankom F57 filter bags with enzymatically undigested residues and 1680 mL of pre-warmed artificial saliva. The pH of the incubation mixture was 6.6 ± 0.1 in both runs. It was measured using a calibrated portable pH meter (Orion Star A221, Thermo Scientific, Montreal, QC, Canada). Cecal degradation was carried out under anaerobic conditions for 48 h, following the experimental approach of Pascual et al. [12] and Tassone et al. [11]. The inoculum was prepared independently for each run from the cecal contents collected.

At the end of cecal incubation, the Ankom F57 filter bags were sequentially rinsed with distilled water and dried at 60 °C for 24 h to remove residual moisture. Following drying, the same allocation of Ankom F57 filter bags used for the determination of pre-cecal enzymatic digestibility was maintained for the determination of residual DM, OM, and CP according to AOAC procedures [13], whereas NDF and ADF were determined according to Van Soest et al. [14] and residual values were corrected using the corresponding blanks. The results obtained after cecal degradation were then used to calculate the cumulative in vitro digestibility (pre-cecal enzymatic + cecal) of DM, OM, CP, NDF, and ADF for each co-product.

#### 2.4.3. Calculation

Digestibility was calculated by mass balance from the initial amount of each component and its blank-corrected residual amount after the corresponding in vitro digestion phases. Calculations were performed for DM, OM, CP, NDF, and ADF.

In vitro pre-cecal enzymatic digestion coefficient was calculated as:

XD _pre-cecal_ (%) = [(X_i_ − X_r, pre-cecal_)/X_i_] × 100

where XD _pre-cecal_ represents the in vitro digestibility coefficient after pre-cecal enzymatic digestion of the component considered (DMD _pre-cecal_, OMD _pre-cecal_, CPD _pre-cecal_, NDFD _pre-cecal_, or ADFD _pre-cecal_), X_i_ is the initial amount of that component, and X_r, pre-cecal_ is its blank-corrected residual amount after the three-step pre-cecal enzymatic digestion.

In vitro pre-cecal enzymatic + cecal digestibility coefficient was calculated as:

XD _pre-cecal+cecal_ (%) = [(X_i_ − X_r, pre-cecal+cecal_)/X_i_] × 100

where XD _pre-cecal+cecal_ represents the cumulative digestibility coefficient after the sequential pre-cecal enzymatic + cecal procedure (DMD _pre-cecal+cecal_, OMD _pre-cecal+cecal_, CPD _pre-cecal+cecal_, NDFD _pre-cecal+cecal_, or ADFD _pre-cecal+cecal_), and X_r, pre-cecal+cecal_ is the blank-corrected residual amount after completion of pre-cecal enzymatic digestion followed by cecal degradation.

In both equations, X represents DM, OM, CP, NDF, or ADF. Initial and residual component amounts were derived from sample or residue DM weight and the corresponding component concentration, with residual values corrected for blanks according to Tassone et al. [11].

The contribution of the cecal phase to nutrient digestibility was calculated as XD _pre-cecal+cecal_ − X_D pre-cecal._ The cecal contribution is expressed in percentage points.

### 2.5. Statistical Analysis

The experiment followed a completely randomized design comprising four treatments, each corresponding to one of the four co-products under evaluation. For chemical composition analyses, each treatment was represented by three independent biological replicates (*n* = 3); analytical replicates were averaged prior to statistical analysis to yield a single value per biological replicate. Data were analyzed by one-way analysis of variance (ANOVA) according to the following model:

$$Y_{ij}=\mu +\tau_{i}+\varepsilon_{ij}$$

where Y_ij_ denotes the response variable, μ the overall mean, τ_i_ the fixed effect of co-products, and ε_ij_ the random residual error.

When the effect of treatment was statistically significant, means were separated using Tukey’s honestly significant difference (HSD) post hoc test. Effects were considered statistically significant at *p* < 0.05.

For the in vitro experiment, the experimental unit was the co-product source within an incubation run (technical replicates averaged), giving 6 observations per variable (3 sources × 2 runs). Data were analyzed with a linear model including co-product, incubation run and their interaction. Run was treated as a fixed blocking factor:

$$Y_{ijk}=\mu +\tau_{i}+R_{j}+{\left(\tau R\right)}_{ij}+\varepsilon_{ijk}$$

where Y_ijk_ is the response of source k of co-product i in run j, μ the overall mean, τ_i_ the fixed effect of co-product, R_j_ the fixed effect of incubation run (blocking factor), (τR)_ij_ the co-product × run interaction and ε_ijk_ the residual error.

Residual normality (Shapiro–Wilk test) and homogeneity of variance (Levene’s test) were assessed. All analyses were performed in R through RStudio (version 2025.05.0 + 496).

To avoid pseudo replication, only independent units were used as replicates in all analyses. For chemical composition, the three independently collected samples of each co-product (biological replicates) were experimental units, and the analytical triplicates were averaged beforehand. For the in vitro experiment, the Ankom F57 bags allocated to the same biological replicate within a jar (*n* = 3 per set) shared the same jar, incubation conditions and inoculum; they were therefore considered technical replicates (subsamples), averaged into a single value per source and run, and not entered in the model as independent observations.

## 3. Results

### 3.1. Chemical Composition

The chemical composition differed significantly among the co-products for all evaluated parameters (*p* < 0.0001; Table 1). The two date-derived co-products had higher DM contents (74.34–80.42% of fresh matter) than pea hulls and artichoke bracts (16.65–17.24% of fresh matter), which did not differ significantly. The CP was highest in pea hulls (24.97% DM), followed by artichoke bracts (16.52% DM), whereas both date flesh and date pits contained considerably lower amounts (approximately 5–6% DM). Conversely, date pits had the highest EE concentration (6.85% DM), while the other co-products contained less than 1.3% DM. Ash concentration was higher in pea hulls and artichoke bracts (6.76 and 6.78% DM, respectively) than in date flesh and date pits (2.39 and 1.78% DM, respectively).

The carbohydrate fractions differed particularly strongly between date flesh and date pits. Date flesh contained the highest NFC concentration (79.45% DM), whereas date pits contained only 4.30% DM. Pea hulls and artichoke bracts showed intermediate and comparable NFC concentrations (19.76 and 20.52% DM, respectively). Conversely, NDF concentration was highest in date pits (81.26% DM) and lowest in date flesh (12.78% DM), with intermediate values in artichoke bracts (54.95% DM) and pea hulls (47.90% DM). A similar pattern was observed for ADF and ADL. Date pits had the highest concentrations of ADF (55.94% DM) and ADL (12.89% DM), whereas date flesh had the lowest values (9.65 and 2.26% DM, respectively). Cellulose concentration was also highest in date pits (43.05% DM) and lowest in date flesh (7.40% DM), while hemicellulose showed a less pronounced differentiation among substrates. Tannin concentrations also differed significantly among the evaluated co-products. Date pits contained the highest concentrations of both condensed tannins (1.52 g CE/100 g DM) and total tannins (2.38 g TAE/100 g DM) whereas pea hulls, artichoke bracts, and date flesh had substantially lower CT concentrations (0.04, 0.02, and 0.08 g CE/100 g DM, respectively) and TT concentrations (0.430, 0.195, and 0.218 g TAE/100 g DM, respectively).

### 3.2. Pre-Cecal Enzymatic Digestibility

The four co-products showed distinct in vitro pre-cecal enzymatic digestibility profiles (Table 2). Date flesh exhibited the highest DMD _pre-cecal_ and OMD _pre-cecal_ values (89.44% and 89.58%, respectively), whereas date pits had the lowest values (29.78% and 29.62%, respectively). Pea hulls and artichoke bracts occupied intermediate pre-cecal enzymatic digestibility with DMD _pre-cecal_ and OMD _pre-cecal_ being significantly higher in pea hulls (54.20% and 54.44%, respectively) than in artichoke bracts (39.57% and 38.59%, respectively).

Date flesh also showed the highest CPD _pre-cecal_ (97.78%), whereas comparable CPD _pre-cecal_ values, around 85%, were observed for pea hulls, artichoke bracts, and date pits.

The pre-cecal enzymatic digestibility of structural carbohydrates showed a different pattern. NDFD _pre-cecal_ was lowest in date pits (21.19%), whereas pea hulls, artichoke bracts, and date flesh showed significantly higher and comparable values, ranging from 30.70 to 35.63%. ADFD _pre-cecal_ did not differ significantly among the four co-products and averaged approximately 20.8%.

### 3.3. Cecal Degradability

Cecal degradability differed significantly among the four co-products (Table 3). Date pits exhibited the highest OMD _cecal_ (17.09%), whereas DMD cecal was comparable among pea hulls, artichoke bracts, and date pits (10.94–14.43%). Artichoke bracts showed the highest CPD _cecal_ (4.16%), while date pits exhibited the lowest value (0.02%). Regarding structural carbohydrates, pea hulls presented the highest NDFD _cecal_ and ADFD _cecal_ (16.48% and 20.83%, respectively), whereas date flesh showed the lowest values (0.05% and 0.73%, respectively).

### 3.4. Cumulative (Pre-Cecal Enzymatic + Cecal) Digestibility

Cumulative digestibility differed significantly among co-products (Table 4). Date flesh exhibited the highest DMD _pre-cecal+cecal_, OMD _pre-cecal+cecal_, and CPD _pre-cecal+cecal_ (89.54%, 89.97%, and 98.53%, respectively), whereas date pits showed the lowest DMD and OMD (41.93% and 46.71%, respectively). Pea hulls presented the highest NDFD _pre-cecal+cecal_ and ADFD _pre-cecal+cecal_ (47.18% and 41.90%, respectively), while date flesh exhibited the lowest ADFD _pre-cecal+cecal_ (22.64%).

## 4. Discussion

The co-products evaluated in the present study differed markedly in chemical composition and digestive behavior, indicating distinct potential roles as energy, protein–fiber, or fibrous ingredients in rabbit diets.

Accordingly, each co-product is first discussed in relation to its nutritional characteristics and potential feeding role, followed by a comparative assessment of pre-cecal enzymatic digestion and the additional contribution of cecal degradation.

### 4.1. Pea Hulls as a Protein–Fiber Resource

Pea hulls combined the highest CP concentration among the evaluated co-products (24.97% DM) with substantial structural fiber (47.90% NDF, 26.66% ADF, and 5.74% ADL), indicating their potential as a protein-containing fibrous ingredient. Sarhan [17] reported a comparable CP concentration (approximately 23.3%) in pea-derived fibrous residues used in growing-rabbit diets, although with lower NDF and ADF concentrations (14.6% and 7.3%, respectively). This difference highlights the compositional variability of pea-processing residues and should be considered when comparing their nutritional value or extrapolating dietary inclusion levels among different materials [17].

The in vitro results further indicated a contribution of cecal degradation. DMD _pre-cecal_ and OMD _pre-cecal_ were 54.20% and 54.44%, respectively, and increased by 10.94 and 11.57 percentage points during the subsequent cecal phase.

The additional contribution was particularly evident for the structural carbohydrate fractions, with increases of 16.48 percentage points for NDFD _pre-cecal+cecal_ and 20.83 percentage points for ADFD _pre-cecal+cecal_, whereas CPD _pre-cecal+cecal_ increased by only 1.70 percentage points. Thus, cecal degradation accounted mainly for the additional digestibility of the residual fibrous fraction.

Rabbit-specific in vivo evidence is provided by Sarhan [17], who evaluated pea vine hay and pea pod hulls as partial or complete replacements for clover hay in growing rabbit diets. Apparent DM digestibility ranged from 61.64% to 64.97%, while crude-fiber digestibility ranged from 33.10% to 40.53%. Although these values refer to complete diets and to pea-derived materials compositionally different from the isolated pea hulls evaluated here, they provide complementary in vivo evidence of the digestive utilization of pea-processing residues. Sarhan [17] also reported favorable nutritive value for the diet containing 30% pea pod hulls, supporting their potential use within the fibrous component of rabbit diets.

From a feed-formulation perspective, pea hulls combine a substantial structural-fiber fraction with a relevant protein contribution. Among conventional feed ingredients used in rabbit diets, dehydrated alfalfa represents a protein-containing fibrous source, generally providing approximately 17–20% CP together with substantial structural fiber, whereas soybean meal is primarily used as a concentrated protein source, typically containing more than 40% CP and considerably less structural fiber [18]. The pea hulls evaluated here (24.97% CP and 47.90% NDF) therefore provide a higher protein contribution than dehydrated alfalfa while retaining a pronounced fibrous component.

Beyond chemical composition, the nutritional value of these feed resources also depends on nutrient digestibility. In rabbits, Villamide et al. [19] reported marked differences in nitrogen and amino-acid digestibility among conventional feedstuffs, including soybean meal and alfalfa meal, as well as peas, indicating that protein utilization varies according to feed source. Similarly, using a three-step enzymatic procedure, Villamide et al. [20] reported an in vitro DM digestibility of approximately 60% for dehydrated alfalfa, compared with 85–90% for protein and energy concentrates. Although these coefficients are not directly comparable with those obtained in the present study, these findings indicate that both nutrient composition and digestive accessibility should be considered when assessing the feeding value of pea hulls.

Accordingly, pea hulls should be regarded primarily as a protein-containing fibrous resource rather than as a direct substitute for soybean meals or other concentrated protein sources. Their relatively high CP concentration may complement their contribution to dietary fiber, but any replacement of conventional ingredients should be based on the nutritional balance of the complete diet, including structural fiber and digestible protein and amino-acid supply. Given the compositional variability of pea-processing residues reported by Sarhan [17], specific in vivo dose–response studies with the present material are required before appropriate substitution and inclusion levels can be established.

### 4.2. Artichoke Bracts as a Potential Fibrous Ingredient

Artichoke bracts are characterized by a high-fibrous profile, containing 54.95% NDF, 36.40% ADF, and 7.89% ADL and a moderate CP concentration (16.52% DM).

The fiber concentrations obtained in the present study are comparable with those reported by Abid [21] for NDF (59.4%), ADF (44.2%) and ADL (10.6%) and Dabbou et al. [22] for NDF (57.4%) and ADF (40.0%) confirming the substantial fibrous character of this co-product. The CP concentration obtained in the present study is higher than the CP concentration reported by Dabbou et al. (10.2%) [22], but slightly lower than that reported by Abid (18.8%) [21]. These differences may be related to differences in cultivar or processing conditions.

The digestive response indicated a substantial contribution of the cecal phase. DMD _pre-cecal_ and OMD _pre-cecal_ were 39.57% and 38.59%, respectively, and increased by 14.43 and 14.72 percentage points after cecal degradation. Additional digestibility was also evident for NDF (10.32 percentage points) and ADF (15.51 percentage points), while CP digestibility increased by 4.16 percentage points. Thus, a relevant proportion of the fibrous fraction remaining after pre-cecal enzymatic digestion was available for subsequent microbial utilization. The present findings can be read alongside the in vivo study of Dabbou et al. [22] in which dried artichoke bracts were included at 50 and 100 g/kg in the diets of 120 growing rabbits, with no adverse effects on final body weight, weight gain, or feed intake. The 50 g/kg inclusion level resulted in the highest digestible energy and favorable apparent digestibility for DM, OM, EE, NDF, and ADF, whereas CP digestibility was not affected.

From a feed-formulation perspective, dehydrated alfalfa represents an appropriate conventional comparator because both ingredients provide substantial structural fiber together with a moderate protein contribution. The nutritional value of fibrous ingredients in rabbits, however, depends not only on fiber concentration but also on the characteristics of the cell-wall fraction. García et al. [23], comparing several fibrous ingredients in rabbits, including alfalfa hay, demonstrated significant differences in cell-wall digestibility and rate of passage according to fiber source. This is relevant to the present results, as the substantial additional disappearance of NDF and ADF from artichoke bracts during cecal degradation indicates that part of their residual structural fraction remained accessible to microbial degradation despite their relatively high fiber concentration.

Overall, the present findings suggest that artichoke bracts may be a candidate fibrous ingredient similar to dehydrated alfalfa. Their high fiber content, moderate protein concentration, and substantial post-cecal disappearance indicate that they can provide both structural and fermentable substrates. Importantly, this potential is consistent with in vivo evidence in growing rabbits, with dried artichoke bracts tested at 5–10% of the diet and the most favorable digestibility response observed at 5% [22]. Nevertheless, the higher CP concentration of the material evaluated in the present study compared with that reported by Dabbou et al. [22] indicates that its specific nutritional contribution should be confirmed in appropriately balanced in vivo diets before an optimal inclusion level is established.

### 4.3. Date Flesh as a Potential Energy-Oriented Ingredient

Date flesh exhibited a carbohydrate-rich composition, with high NFC (79.45% DM) and low NDF, ADF, and ADL concentrations (12.78%, 9.65%, and 2.26%, respectively), confirming its potential as an energy-oriented feed ingredient. This composition is consistent with the predominance of soluble carbohydrates reported for date flesh. Elleuch et al. [24] reported that sucrose, glucose, and fructose accounted for 73–79% DM in Tunisian Deglet-Nour and Allig dates, while Assirey [25] reported total sugar concentrations of 71.2–81.4% DM across ten Saudi Arabian cultivars. The CP concentration observed here (5.22% DM) was slightly higher than those reported in these studies (1.72–4.04% DM), whereas EE (0.17% DM) and ash (2.39% DM) were within or close to published ranges [24,25].

The high availability of this soluble carbohydrate fraction was reflected in its digestive response. DMD _pre-cecal_ and OMD _pre-cecal_ reached 89.44% and 89.58%, respectively, while the subsequent cecal phase produced only minor increases (0.10 and 0.39 percentage points). Similarly, cecal degradation increased CP digestibility by only 0.75 percentage points and produced little additional digestibility of NDF (0.05 percentage points) and ADF (0.73 percentage points). Thus, date flesh appears more suitable as a readily available energy source. Abo-Eid et al. [26] evaluated growing-rabbit diets containing 0–40% date waste meal and reported the most favorable digestibility response at 20% inclusion with enzyme supplementation, with apparent DM, OM, and CP digestibility of 74.66%, 74.91%, and 87.64%, respectively. Although the study evaluated complete diets containing date waste meal rather than isolated date flesh, it provides relevant in vivo evidence of the digestive utilization of date-derived feed resources in rabbits and supports their potential incorporation into nutritionally balanced diets [26].

From a feed-formulation perspective, date flesh may be considered primarily as a potential energy-oriented ingredient. Unlike conventional cereal grains such as maize and barley, in which starch represents the major energy-yielding carbohydrate, date flesh is characterized by a predominance of soluble sugars [18,24,25]. This difference is nutritionally relevant in rabbits, as carbohydrate type and pre-cecal digestibility influence the amount and nature of substrate reaching the cecal microbiota [27]. Date flesh should therefore be regarded as a complementary energy source rather than as a nutritionally equivalent weight-for-weight substitute for cereal grains. Its potential inclusion must also account for its low structural fiber content, particularly the low ADF and ADL concentrations observed in the present study. Adequate levels of structural fiber are required to maintain normal digesta passage and digestive function in growing rabbits [27]. Consequently, the incorporation of date flesh would require appropriate adjustment of fibrous ingredients to maintain the structural fiber balance of the complete diet.

Overall, the high DMD _pre-cecal_ and OMD _pre-cecal_, together with the minimal additional contribution of cecal degradation, indicate that date flesh was predominantly utilized during simulated pre-cecal digestion, supporting its potential role as a readily available energy source. Although available in vivo evidence supports the utilization of date-derived materials in rabbit diets [26], specific dose–response studies with isolated date flesh are required to establish appropriate inclusion levels in nutritionally balanced diets.

### 4.4. Date Pits: Nutritional Constraints Related to Fiber and Tannins

Date pits exhibited the most pronounced fibrous and lignified profile among the evaluated co-products, with NDF, ADF, and ADL concentrations of 81.26%, 55.94%, and 12.89%, respectively. Cellulose accounted for 43.05%, whereas CP and NFC concentrations were comparatively low (5.82% and 4.30% DM, respectively).

The low CP concentration observed in the present study is consistent with previous characterizations of date pits. Bouaziz et al. [28] reported CP concentrations ranging from 5.1 to 5.6% in Deglet Nour and Allig date pits, while Abid et al. [29] reported a CP concentration of 6.0% in Chedakh pits. Similarly, Hamada et al. [30] reported CP concentrations ranging from 5.0 to 6.3% across Fard, Khala, and Lulu pit varieties. The relatively narrow range of CP concentrations reported across these studies suggests that the low protein content is a consistent characteristic of date pits.

The high fiber concentrations observed in the present study are comparable to those of previous studies. Abid et al. [29] reported NDF and ADF concentrations of 67.7% and 31.9%, respectively, in Chedakh pits, whereas Hamada et al. [30] reported NDF concentrations of 65–69% and ADF concentrations of 46–51% across Fard, Khala, and Lulu varieties.

This composition was associated with the lowest pre-cecal digestive utilization among the evaluated co-products and indicates restricted accessibility of the pit matrix to pre-cecal digestion. DMD _pre-cecal_ and OMD _pre-cecal_ were only 29.78% and 29.62%, respectively, with NDFD _pre-cecal_ reaching 21.19%. Nevertheless, the subsequent cecal phase produced appreciable additional digestibility of DM (12.15 percentage points), OM (17.09), NDF (12.75), and ADF (13.10). Thus, cecal degradation contributed appreciably to the utilization of the fraction remaining after pre-cecal enzymatic digestion, although cumulative digestibility remained comparatively low. The limited digestive accessibility of date pits is consistent with their highly fibrous and lignified matrix, as lignification can restrict microbial degradation of structural carbohydrates in rabbits [27]. However, fiber utilization also depends on cell-wall characteristics and accessibility. In growing rabbits, Carrión et al. [31] reported an in vivo NDF digestibility of 43.0% for palm pit meal, demonstrating that fibrous co-products can differ markedly in fiber utilization. Although not directly comparable with the present in vitro results, this finding supports the importance of fiber characteristics, rather than concentration alone, in determining digestive utilization.

Date pits were also distinguished from the other evaluated co-products by their markedly higher concentrations of condensed (1.52 g CE/100 g DM) and total tannins (2.38 g TAE/100 g DM). Tannins can interact with proteins and cell-wall components, potentially reducing nutrient accessibility, although their effects depend on concentration, chemical structure, and dietary matrix [32]. Studies on date-processing co-products have also indicated that tannins may influence in vitro fermentation, as shown by the use of polyethylene glycol to assess tannin-related effects on the fermentative response of date-derived substrates [33]. In rabbits, Yang et al. [34] reported that dietary supplementation with tannic acid at 0.75–3 g/kg did not significantly affect nutrient digestibility, although the highest level reduced feed intake and altered cecal degradation and microbial composition. These findings indicate that the nutritional effects of tannins in rabbits are dose- and response-dependent; however, their relevance to date pits should also consider the naturally occurring tannin forms and their interactions within the fibrous seed matrix.

Overall, the limited digestive utilization of date pits likely reflects the combined influence of their highly fibrous and lignified matrix and high tannin content rather than the effect of a single component. The structural characteristics of the seed matrix may limit enzymatic accessibility, while tannins may further constrain nutrient availability through interactions with proteins and cell-wall constituents. Nevertheless, the relative contributions of these factors cannot be determined from the present results and require further investigation. Feeding studies in rabbits have reported variable responses to dietary date pits. Aboul-Ela et al. [35] observed reduced growth and feed efficiency when ground date pits were included as a partial replacement for maize, whereas Ibrahim et al. [36] reported improved feed efficiency when ground date pits partially replaced maize, with enzyme supplementation producing a modest improvement in nutrient digestibility. Similarly, Mousa [37] evaluated ground date pits as a partial replacement for barley grain in growing-rabbit diets and reported significant effects on nutrient digestibility and productive performance, further indicating that their feeding value depends on dietary inclusion and formulation. More recently, Shuaibu et al. [38] evaluated roasted date palm seed meal at inclusion levels of 0–20% in weaned rabbits and reported significant effects on growth and nutrient digestibility, with effective utilization up to the highest inclusion level tested.

Despite their relatively high EE concentration (6.85% DM), the low digestive accessibility observed in the present study suggests limited potential for untreated date pits as a direct substitute for conventional feed ingredients. The contrasting responses reported in rabbits, together with the results obtained with enzyme-supplemented ground date pits [36] and roasted date palm seed meal [38], indicate that processing and dietary formulation may substantially influence their nutritional utilization. Specific in vivo dose–response studies are therefore required to determine whether appropriate processing can improve the feeding value of the date pits evaluated here and to establish suitable inclusion levels in nutritionally balanced rabbit diets.

### 4.5. Comparative Contribution of Pre-Cecal Enzymatic Digestion and Cecal Degradation

The differences among the four co-products were reflected not only in pre-cecal enzymatic digestibility but also in the extent to which the residual fractions were utilized during the subsequent cecal phase. This comparison is relevant because in vitro evaluation of rabbit feeds has traditionally used multienzyme, cecal-inoculum, or fecal-inoculum procedures as separate analytical approaches. Pascual et al. [12] reported higher DMD estimates with the multienzyme method than with inoculum-based procedures, although both multienzyme and cecal estimates were strongly related to in vivo DMD (R^2^ = 0.95 and 0.88, respectively). Tassone et al. [11] similarly found higher estimates and greater reproducibility with a multienzyme procedure than with fecal-inoculum methods using the Ankom Daisy^II^ system.

In contrast to these comparative approaches, the present study applied pre-cecal enzymatic digestion and cecal degradation sequentially, with the latter performed on the undigested enzymatic residue. This design distinguishes pre-cecal enzymatic digestibility during simulated pre-cecal digestion from the additional microbial utilization measured after cecal degradation, thereby providing an integrated assessment of the digestive fate of each substrate.

The relevance of substrate pre-digestion has been demonstrated experimentally in rabbits. Ocasio-Vega et al. [39] compared carbohydrate-rich substrates incubated with rabbit soft-fecal inoculum with or without prior pepsin–pancreatin digestion. Pre-digestion modified fermentation kinetics and volatile fatty acid production in a substrate- and donor-diet-dependent manner, leading the authors to recommend pre-digestion before in vitro cecal fermentation. These findings support the evaluation of microbial fermentation on substrates previously subjected to simulated pre-cecal digestion.

Substrate characteristics are also major determinants of cecal fermentation. Lavrenčič [40] compared starch, pectin, xylan, cellulose, a commercial rabbit feed, and its isolated NDF using rabbit cecal inoculum. During the first 10 h of incubation, corresponding approximately to the normal cecal retention time considered in that study, pectin and the complete feed were fermented rapidly, whereas cellulose showed substantially slower fermentation. This indicates that microbial utilization is strongly influenced by the physicochemical characteristics and accessibility of the carbohydrate fraction rather than by its concentration alone.

The magnitude of the cecal contribution varied among the evaluated substrates. The cecal phase increased DMD _pre-cecal+cecal_ and OMD _pre-cecal+cecal_ by only 0.10 and 0.39 percentage points in date flesh, compared with 10.94 and 11.57 points in pea hulls, 14.43 and 14.72 points in artichoke bracts, and 12.15 and 17.09 points in date pits. Thus, date flesh was utilized predominantly during pre-cecal enzymatic digestion, whereas the other substrates retained substantially more material available for subsequent microbial degradation.

The contribution of cecal degradation was particularly evident for the structural carbohydrate fractions. After pre-cecal enzymatic digestion, NDFD _pre-cecal_ was comparable among pea hulls, artichoke bracts, and date flesh, while date pits showed the lowest value. Cecal degradation subsequently increased NDF digestibility by 16.48, 10.32, and 12.75 percentage points in pea hulls, artichoke bracts, and date pits, respectively, while the increase in date flesh was negligible (0.05 percentage points). A similar pattern was observed for ADFD. Although ADFD _pre-cecal_ did not differ significantly among the four substrates (19.13–21.91%), the additional contribution of cecal degradation varied markedly, reaching 20.83 percentage points in pea hulls, 15.51 in artichoke bracts, and 13.10 in date pits, compared with only 0.73 in date flesh. These results indicate that the enzymatic phase alone may underestimate the digestibility of fibrous co-products and highlight the importance of accounting for post-cecal microbial fermentation when evaluating their fiber utilization. In contrast, the additional contribution of cecal degradation to CP digestibility was comparatively limited by 0.75, 1.70, and 4.16 percentage points in date flesh, pea hulls, and artichoke bracts, respectively, while date pits remained unchanged. Thus, the additional contribution of cecal degradation depended on both substrate characteristics and the nutrient fraction considered, with a more pronounced contribution to structural carbohydrate degradation than to CP digestibility. Consequently, assessments based solely on pre-cecal enzymatic digestion may underestimate the digestibility of fibrous co-products by failing to account for the microbial degradation of residual cell-wall components. Incorporating the cecal phase therefore provides a more comprehensive assessment of fiber digestibility and highlights the importance of microbial fermentation in the utilization of co-products with contrasting compositional characteristics.

A similar need to integrate chemical composition, digestion, and fermentation has been reported for heterogeneous rabbit-feed resources. Ferreira et al. [41] evaluated 16 conventional and unconventional ingredients and identified markedly different digestive and fermentative profiles. Their findings support the combined interpretation of compositional and microbial measurements, particularly for fibrous ingredients whose nutritional value may be underestimated by enzymatic digestibility alone.

The source of microbial inoculum also affects in vitro estimates. Aderibigbe and Cheeke [42] reported different degradation patterns when rabbit cecal contents and bovine rumen fluid were used to incubate feed ingredients, with slower and generally lower disappearance using rabbit cecal inoculum. Within rabbit-derived inocula, Bovera et al. [43] compared cecal contents and hard feces using dehydrated alfalfa, beet pulp, and barley and found inoculum-dependent differences in fermentation parameters, although substrate rankings were broadly preserved. These studies emphasize the importance of inoculum source when interpreting microbial degradation and support the use of rabbit cecal contents when the objective is to characterize fermentation associated specifically with the cecum.

Overall, the sequential approach differentiated substrates whose digestive utilization occurred predominantly during enzymatic digestion from those for which cecal microorganisms made a substantial additional contribution, particularly to residual structural carbohydrates. This sequential assessment therefore provides a more comprehensive characterization of digestive utilization than either phase considered separately. However, further studies combining the sequential in vitro procedure with parallel in vivo digestibility measurements would be useful to assess its ability to reflect the overall digestive utilization occurring in rabbits and investigate fermentation parameters, including pH, volatile fatty acid concentrations, and gas production.

### 4.6. Limitations of the In Vitro Approach

The results of this study should be interpreted as in vitro estimates and not as direct measures of in vivo digestibility. First, digestibility was calculated from the disappearance of DM, OM, CP, NDF and ADF from Ankom F57 filter bags. With a nominal pore size of 25 µm, these bags allow the loss of soluble compounds and fine particles, which are recorded as disappeared even if they were not enzymatically or microbially degraded. This may have overestimated DM, OM and CP disappearance, particularly for materials rich in soluble components such as date flesh, and the proportion of material lost by solubilization versus true degradation could not be separated in the present study. Second, the procedure cannot reproduce several features of rabbit digestion, including digesta passage, the colonic separation mechanism, the absorption of fermentation products and cecotrophy.

## 5. Conclusions

Pea hulls, artichoke bracts, date flesh, and date pits showed distinct nutritional and digestive profiles. Date flesh showed the highest in vitro digestibility and an energy-rich composition, mostly during simulated pre-cecal digestion. Pea hulls (protein–fiber resource) and artichoke bracts (fibrous ingredient) showed greater additional degradation of structural carbohydrates during cecal degradation. Date pits, highly lignified and tannin-rich, had the lowest in vitro digestibility, only partly improved by cecal degradation, and may require processing before inclusion in rabbit diets. The sequential in vitro approach gave a more complete characterization than a single digestion phase. However, in vivo trials are needed to validate these results.

## Figures and Tables

**Table 1:** Chemical composition of co-products.

Item	Pea Hulls	Artichoke Bracts	Date Flesh	Date Pits	SEM	*p*-Value
DM ^1^	16.65 ^b^	17.24 ^b^	74.34 ^a^	80.42 ^a^	5.750	<0.0001
CP	24.97 ^a^	16.52 ^b^	5.22 ^c^	5.82 ^c^	0.4755	<0.0001
EE	0.61 ^bc^	1.22 ^b^	0.17 ^c^	6.85 ^a^	0.456	<0.0001
Ash	6.76 ^a^	6.78 ^a^	2.39 ^b^	1.78 ^b^	0.778	<0.0001
NFC	19.76 ^b^	20.52 ^b^	79.45 ^a^	4.30 ^c^	3.510	<0.0001
NDF	47.90 ^c^	54.95 ^b^	12.78 ^d^	81.26 ^a^	3.707	<0.0001
ADF	26.66 ^c^	36.40 ^b^	9.65 ^d^	55.94 ^a^	2.417	<0.0001
ADL	5.74 ^c^	7.89 ^b^	2.26 ^d^	12.89 ^a^	1.112	<0.0001
HC	21.24 ^ab^	18.55 ^b^	3.12 ^c^	25.32 ^a^	2.590	<0.0001
CEL	20.92 ^c^	28.51 ^b^	7.40 ^d^	43.05 ^a^	2.510	<0.0001
CT ^2^	0.04 ^b^	0.02 ^b^	0.08 ^b^	1.52 ^a^	0.1171	<0.0001
TT ^3^	0.430 ^b^	0.195 ^c^	0.218 ^c^	2.38 ^a^	1.400	<0.0001

Values are expressed as means (*n* = 3) replicates per co-product. DM, dry matter; CP, crude protein; EE, ether extract; NFC, non-fibrous carbohydrates; NDF, neutral detergent fiber; ADF, acid detergent fiber; ADL, acid detergent lignin; HC, hemicellulose; CEL, cellulose; CT, condensed tannin; TT, total tannin; SEM, standard error of the mean. ^1^ DM is expressed as a % of fresh matter; ^2^ CT are expressed as g CE/100 g DM and ^3^ TT are expressed as g TAE/100 g DM, whereas all other components are expressed as a % of DM. ^a–d^ Means within a row with different superscript letters differ significantly according to Tukey’s multiple-comparison test (*p* < 0.05).

**Table 2:** In vitro pre-cecal enzymatic digestibility coefficients of co-products (%).

Item	Pea Hulls	Artichoke Bracts	Date Flesh	Date Pits	SEM	*p*-Value
DMD _pre-cecal_	54.20 ^b^	39.57 ^c^	89.44 ^a^	29.78 ^d^	3.750	<0.0001
OMD _pre-cecal_	54.44 ^b^	38.59 ^c^	89.58 ^a^	29.62 ^d^	4.083	<0.0001
CPD _pre-cecal_	85.13 ^b^	85.70 ^b^	97.78 ^a^	85.48 ^b^	3.121	<0.0001
NDFD _pre-cecal_	30.70 ^a^	31.16 ^a^	35.63 ^a^	21.19 ^b^	3.170	<0.0001
ADFD _pre-cecal_	21.07	21.12	21.91	19.13	3.500	0.9658

Values are expressed as means (*n* = 6 replicates per co-product). DMD _pre-cecal_, dry matter pre-cecal enzymatic digestibility coefficient; OMD _pre-cecal_, organic matter pre-cecal enzymatic digestibility coefficient; CPD _pre-cecal_, crude protein pre-cecal enzymatic digestibility coefficient; NDFD _pre-cecal_, neutral detergent fiber pre-cecal enzymatic digestibility coefficient; ADFD _pre-cecal_, acid detergent fiber pre-cecal enzymatic digestibility coefficient; SEM, standard error of the mean. ^a–d^ Means within a row with different superscript letters differ significantly according to Tukey’s multiple-comparison test (*p* < 0.05). The absence of superscript letters indicates no significant differences among co-products.

**Table 3:** In vitro cecal degradability coefficients of co-products (%).

Item	Pea Hulls	Artichoke Bracts	Date Flesh	Date Pits	SEM	*p*-Value
DMD _cecal_	10.94 ^a^	14.43 ^a^	0.10 ^b^	12.15 ^a^	2.511	<0.0001
OMD _cecal_	11.57 ^b^	14.72 ^b^	0.39 ^c^	17.09 ^a^	2.321	<0.0001
CPD _cecal_	1.70 ^b^	4.16 ^a^	0.75 ^bc^	0.02 ^c^	0.831	<0.0001
NDFD _cecal_	16.48 ^a^	10.32 ^b^	0.05 ^c^	12.75 ^ab^	3.24	<0.0001
ADFD _cecal_	20.83 ^a^	15.51 ^b^	0.73 ^c^	13.10 ^b^	2.91	<0.0001

Values are expressed as means (*n* = 6 replicates per co-product). DMD cecal, dry matter cecal degradability coefficient; OMD cecal, organic matter cecal degradability coefficient; CPD cecal, crude protein cecal degradability coefficient; NDFD cecal, neutral detergent fiber cecal degradability coefficient; ADFD cecal, acid detergent fiber cecal degradability coefficient; SEM, standard error of the mean. ^a–c^ Means within the same row bearing different superscript letters differ significantly according to Tukey’s multiple-comparison test (*p* < 0.05).

**Table 4:** In vitro cumulative digestibility coefficients of co-products (%).

Item	Pea Hulls	Artichoke Bracts	Date Flesh	Date Pits	SEM	*p*-Value
DMD _pre-cecal+cecal_	65.14 ^b^	54.00 ^c^	89.54 ^a^	41.93 ^d^	3.75	<0.0001
OMD _pre-cecal+cecal_	66.01 ^b^	53.31 ^c^	89.97 ^a^	46.71 ^d^	3.12	<0.0001
CPD _pre-cecal+cecal_	86.83 ^b^	89.86 ^b^	98.53 ^a^	85.50 ^b^	5.97	<0.0001
NDFD _pre-cecal+cecal_	47.18 ^a^	41.48 ^ab^	35.68 ^bc^	33.94 ^c^	6.94	0.0002
ADFD _pre-cecal+cecal_	41.90 ^a^	36.63 ^ab^	22.64 ^c^	32.23 ^b^	5.91	<0.0001

Values are expressed as means (*n* = 6 replicates per co-product). DMD _pre-cecal+cecal_, dry matter pre-cecal + cecal digestibility coefficient; OMD _pre-cecal+cecal_, organic matter pre-cecal + cecal digestibility coefficient; CPD _pre-cecal+cecal_, crude protein pre-cecal + cecal digestibility coefficient; NDFD _pre-cecal+cecal_, neutral detergent fiber pre-cecal + cecal digestibility coefficient; ADFD _pre-cecal+cecal_, acid detergent fiber pre-cecal + cecal digestibility coefficient; SEM, standard error of the mean. ^a–d^ Means within the same row bearing different superscript letters differ significantly according to Tukey’s multiple-comparison test (*p* < 0.05).

## Data Availability

Data is available from the corresponding author upon reasonable request.

## Abbreviations

The following abbreviations are used in this manuscript:

ADF	Acid detergent fiber
ADFD _cecal_	Acid detergent fiber cecal enzymatic degradation coefficient
ADFD _pre-cecal_	Acid detergent fiber pre-cecal enzymatic digestibility coefficient
ADFD _pre-cecal+cecal_	Cumulative acid detergent fiber enzymatic + cecal digestibility coefficient
ADL	Acid detergent lignin
ANOVA	Analysis of variance
AOAC	Association of Official Analytical Chemists
CE	Catechin equivalents
CEL	Cellulose
CP	Crude protein
CPD _cecal_	Crude protein cecal degradation coefficient
CPD _pre-cecal_	Crude protein pre-cecal enzymatic digestibility coefficient
CPD _pre-cecal+cecal_	Cumulative crude protein enzymatic + cecal digestibility coefficient
CT	Condensed tannin
DM	Dry matter
DMD _cecal_	Dry matter cecal degradation coefficient
DMD _pre-cecal_	Dry matter pre-cecal enzymatic digestibility coefficient
DMD _pre-cecal+cecal_	Cumulative dry matter enzymatic + cecal digestibility coefficient
EE	Ether extract
FAO	Food and Agriculture Organization of the United Nations
HC	Hemicellulose
NDF	Neutral detergent fiber
NDFD _cecal_	Neutral detergent fiber cecal degradation coefficient
NDFD _pre-cecal_	Neutral detergent fiber pre-cecal enzymatic digestibility coefficient
NDFD _pre-cecal+cecal_	Cumulative neutral detergent fiber pre-cecal enzymatic + cecal digestibility coefficient
NFC	Non-fibrous carbohydrates
OM	Organic matter
OMD _pre-cecal_	Organic matter pre-cecal enzymatic digestibility coefficient
OMD _pre-cecal+cecal_	Cumulative organic matter pre-cecal enzymatic + cecal digestibility coefficient
SEM	Standard error of the mean
TT	Total tannin
TAE	Tannic acid equivalents
